# Editorial: Novel Biomarkers for Type 2 Diabetes

**DOI:** 10.3389/fendo.2019.00649

**Published:** 2019-09-27

**Authors:** Tarunveer S. Ahluwalia, Tuomas O. Kilpeläinen, Sandeep Singh, Peter Rossing

**Affiliations:** ^1^Steno Diabetes Center Copenhagen, Gentofte, Denmark; ^2^Novo Nordisk Foundation Center for Basic Metabolic Research, Faculty of Health and Medical Sciences, University of Copenhagen, Copenhagen, Denmark; ^3^Department of Human Genetics and Molecular Medicine, Central University of Punjab, Bathinda, India; ^4^Department of Clinical Medicine, University of Copenhagen, Copenhagen, Denmark

**Keywords:** type 2 diabetes, biomarker, omics, data science, personalized medicine

Diabetes Mellitus, commonly known as diabetes, is a complex metabolic disorder characterized by hyperglycemia over a prolonged period. The prevalence of diabetes is spiraling globally with an estimated 425 million adults having diabetes in 2017 and 629 million by 2045 (1). In the year 2017 alone, diabetes caused an estimated 4 million deaths and a global economic health related expenditure of ~730 billion US$ (1). Diabetes, if untreated, may lead to a number of serious chronic complications involving smaller vessels, i.e., microvascular complications, and larger vessels, i.e., macrovascular complications. The microvascular complications involve the kidney, with chronic kidney disease (nephropathy) being the most prevalent cause of end-stage kidney disease globally, and nerve damage (neuropathy) that increases the risk of diabetic foot ulcers and/or amputations, the most expensive complication of diabetes. Furthermore, damage to eyes (retinopathy), may lead to blindness. The most common cause of mortality in diabetes are, however, macrovascular complications that include cardiovascular disease and stroke (1).

Albeit there are different forms of diabetes, Type 2 Diabetes (T2D) is the most common type (~90%) and is characterized by insulin resistance, i.e., the body's inability to respond fully to insulin, that may lead to decreased insulin production. T2D is an excellent example of a disease in which the etiology is characterized by gene-environment interaction (GEI) where modern-day sedentary lifestyle along with genetic susceptibility predisposes the individual to an increased risk of developing diabetes. In the past decade, large scale genome wide association studies (GWAS) (2) and GEI studies (3) laid the foundations for omics era by identifying hundreds of genetic risk variants that in aggregate explain a substantial part of the heritability of T2D. More recently, exome sequencing (4) and exome wide association studies (5, 6), enriched for missense and low frequency variants, have discovered additional genes and pathways associated with risk for T2D and its complications (7). We have recently identified a number of promising blood and urine-based biomarkers of inflammatory, endothelial, and microbiome origin (8), for diabetic complications, especially diabetic kidney disease (DKD) (9, 10). However, identifying biomarkers for T2D and its complications remains challenging due to the heterogeneous nature of T2D. The heterogeneity relates not only to glycemic control or treatment response (11), but also to clinical phenotypes such as age of onset and body mass index, biochemical characteristics such as insulin resistance, and differences in environmental exposures, which all lead to disease variability (12). Some recent studies using data clustering methods have demonstrated that diabetes may be clustered into smaller, somewhat homogenous subgroups using individual-level clinical, genetic and metabolic data (13, 14), which suggests that there is need for developing stratified treatments. In line with these observations, the current issue highlights recent research articles focusing on biomarkers for type 2 diabetes and its complications.

Winter et al. provide an overview of inflammatory markers involved in the activation of innate and adaptive immune systems that are recognized as key mediators for the development and progression of renal damage in DKD. The authors discuss a hypothesis whereby the activation of inflammatory pathways leads to a state of chronic systemic inflammation and diabetic complications, in particular atherosclerosis and CVD, in individuals with T2D, and provide evidence on the potential of anti-inflammatory therapies in reducing the risk of diabetes complications. Winter et al. discusses cross sectional data on acute phase proteins (C-reactive protein or hsCRP and fibrinogen) and pro-inflammatory cytokines (IL-6 and TNF-α) that associate with increased risk of T2D. After adjustment for body mass index and adiposity traits, the association between hsCRP and T2D is attenuated (Winter et al.). This highlights adipose tissue-mediated low-grade inflammation being associated with increased insulin resistance and T2D development, especially in obese individuals (15). Obesity, one of the major contributors to T2D pathogenesis, leads to rapid fat cell expansion in both cell size and number, with their oxygen demand exceeding the supply. This low-oxygen state, hypoxia, leads to upregulation of the anti-hypoxic protein HIF-1α, which in turn causes tissue inflammation and prevents fat cells (adipocytes) from responding normally to insulin (16, 17). A meta-analysis of GWAS on hsCRP showed shared genes and pathways that are associated with insulin resistance and T2D (18, 19), consistent with another study identifying body fat percentage genes linking adiposity and cardio-metabolic risk (20). Finally, Winter et al. discuss the kidney's vulnerability to complement-mediated injury, and potential biomarker associations with different kidney phenotypes (glomerulonephritis, tubulointerstitial injury, C3 glomerulopathy). Interestingly, the authors underscore the role of leukocytes in renal complications and highlight neutrophil-lymphocyte ratio as a potential biomarker for DKD.

Yim et al. report a blood-based biomarker nertrin-1, a laminin-related protein whose levels are increased in individuals with impaired fasting glucose (IFG) and T2D. Netrin 1 promotes leukocyte migration in peripheral organs, tissue regeneration, and modulation of inflammation, which may explain it relevant to T2D as discussed above. Moreover, netrin-1 exhibits an anti-angiogenic effect, allowing higher blood flow to hypoxic tissue, and it has demonstrated cardio-protective effects by preventing ischemia-reperfusion injury in animal studies (Yim et al.). Although the study is interesting, observing positive correlations between nertrin-1 and insulin resistance and T2D, and a negative correlation with estimated glomerular filtration rate (eGFR) (a proxy of kidney function measure), in cross-sectional data, the authors underscore the need for longitudinal studies and validation studies—a highly relevant consideration for any biomarker study.

Vaishya et al. highlight the relevance of developing new biomarkers—proteins, metabolites, MicroRNA or other biomolecules—for T2D to complement traditional biomarkers such as HbA1c and blood glucose. By definition, a biomarker is a biomolecule/biological state that can be used for the prognosis, diagnosis, and follow-up of the pathological state or the severity of a disease. The current biomarkers used in the clinic (HbA1c, plasma glucose etc.) are only useful after the manifestation of the disease and fail to completely account for heterogeneity in diabetes pathogenesis, insulin resistance and insulin production, and thus do not guide toward the right choice of therapy. In addition, there is a need for developing biomarkers to early and precise prediction of diabetes complications. Thus, the identification of more specific, stage-related, non-invasive biomarkers with a greater accuracy in diagnosis and progression of T2D is vital. The authors also discuss the relevance of emerging biomarkers such as microRNAs, proteins and metabolites, listing some of the most relevant biomarker candidates associated with diabetes and its complications. The authors discuss very important issues relating to improving individual risk assessment methods with addition of multi-marker approaches and models comprising longitudinal data points. Albeit classic GWAS and recent metabolomics and proteomics approaches involving large consortia or biobanks (3, 5, 21), are required for biomarker discovery (6), more sophisticated modeling, longitudinal study designs, and data integration approaches are essential for the validation of these biomolecules as relevant biomarkers for T2D.

Recent studies have highlighted brain as a key player in glucose regulation and the pathogenesis of metabolic disorders such as T2D (22). Liang et al. propose that chronic psychological stress plays an important role in T2D pathogenesis and use microRNAs as biomarkers measuring these surrogate phenotypes. There is evidence suggesting that increased activation of hypothalamus-pituitary-adrenal (HPA) axis and sympathetic nervous system (SNS), and consequent elevation of stress hormones, may be important for the etiology and development of T2D. Liang et al. explore the association between neuroendocrine stress response-related circulating miRNAs and T2D and identify a number of interesting associations that may point toward potential biomarkers for prediabetes and insulin resistance in adults.

Different omics platforms—genomics, metabolomics, proteomics, and microbiomics—and RNA sequencing-based studies, coupled to novel data science methods involving bioinformatics, data mining, imaging, machine learning, neural networks, are now revolutionizing biomarker development (21, 23, 24). Specialized health care centers, diabetes clinics and hospitals that manage patient-based registers longitudinally and impart education to patients and health workers, have already started effective collaborations with data scientists and/or research field specialists to narrow the gap between bio-banking, biomarker development, and biomarker validation in intervention studies or clinical trials. Adopting such methods, in accordance with data protection and ethical guidelines, with emphasis on judicial use of follow up patient data is steadily moving closer to effective personalized treatment (23) and the building of efficient translational research facilities, which promises to improve the quality of life for the patients and enable better health care.

## Author Contributions

TA has written the first draft of the editorial. All authors listed have made a substantial, direct and intellectual contribution to the work, and approved it for publication.

### Conflict of Interest

The authors declare that the research was conducted in the absence of any commercial or financial relationships that could be construed as a potential conflict of interest.
